# Application and clinical impact of the RESIST-4 O.K.N.V. rapid diagnostic test for carbapenemase detection in blood cultures and clinical samples

**DOI:** 10.1007/s10096-020-04021-4

**Published:** 2020-09-07

**Authors:** Sophie Roth, Fabian K. Berger, Andreas Link, Anna Nimmesgern, Philipp M. Lepper, Niels Murawski, Jörg T. Bittenbring, Sören L. Becker

**Affiliations:** 1 Institute of Medical Microbiology and Hygiene, Saarland University, Homburg, Germany; 2 Department of Internal Medicine III, Saarland University, Homburg, Germany; 3 Department of Internal Medicine V, Saarland University, Homburg, Germany; 4 Department of Internal Medicine I, Saarland University, Homburg, Germany

**Keywords:** Antibiotics, Clinical microbiology, Diagnostic stewardship, *Enterobacterales*, *Escherichia coli*, *Klebsiella pneumoniae*, Treatment

## Abstract

Invasive infections caused by carbapenemase-producing bacteria are associated with excess mortality. We applied a rapid diagnostic test (RDT) on clinical samples with an elevated likelihood of carbapenemase-producing bacteria and documented its impact on antibiotic treatment decisions. Among 38 patients, twelve tested positive for infections caused by carbapenemase-producing bacteria (31.6%), mainly in blood cultures. KPC (*n* = 10) was more frequent than OXA-48 (*n* = 2). RDT-based carbapenemase detection led to a treatment modification to ceftazidime/avibactam-containing regimens in all patients before detailed antibiotic testing results became available. Eleven patients (92%) survived the acute infection, whereas one patient with a ceftazidime/avibactam- and colistin-resistant OXA-48-positive isolate died.

## Introduction

Increasing antimicrobial resistance is among the biggest threats to global health. Resistance in gram-negative bacteria is particularly worrying, e.g. *Enterobacterales* and non-fermentative bacteria that produce carbapenemases, leading to non-susceptibility to all carbapenem antibiotics. Carbapenemase-carrying plasmids are easily transferred to other bacteria through horizontal gene transfer. A significant increase in carbapenemase-producing bacteria such as *Klebsiella pneumoniae* in high-income countries is associated with an excess mortality of up to 50% [1], with inadequate empirical treatment and delayed diagnosis of carbapenem resistance being major contributing factors [2, 3]. It is pivotal to reduce the time to appropriate antimicrobial treatment by early detection of multiresistant pathogens and to elucidate the underlying resistance mechanism to switch treatment, e.g. to new beta-lactam/beta-lactamase inhibitor combination antibiotics such as ceftazidime/avibactam or the siderophore cephalosporin cefiderocol with a broad activity against frequently isolated carbapenemases in Europe, i.e. KPC and OXA-48 [4].

Recently, immunochromatographic rapid diagnostic tests (RDTs) to detect different carbapenemases in culture-grown bacteria within 10–20 min have been introduced. In the present study, we investigated the diagnostic accuracy and subsequent impact on clinical decision-making of the RESIST-4 O.K.N.V. test, an RDT for carbapenemase detection in clinical samples.

## Methods

This study was conducted at the Saarland University Medical Center, a tertiary care centre in Homburg, Germany. We evaluated the microbiological characteristics and clinical impact of the RESIST-4 O.K.N.V. RDT (Coris BioConcept; Gembloux, Belgium) for rapid carbapenemase detection and compared it to a commercially available multiplex PCR test (RDB2290 Carbapenemase*,* Autoimmun Diagnostika GmBH; Straßberg, Germany). The RDT was previously validated for application on culture-grown bacterial colonies and uses specific monoclonal antibodies directed against OXA-48, NDM, KPC and VIM-1 carbapenemases. The test was performed according to the manufacturer’s instructions [5].

In the first phase of this study (30 April 2018–30 April 2019), the RDT was applied on clinical samples with growth of gram-negative bacteria and decreased susceptibility to ertapenem, imipenem and/or meropenem, as detected by automated resistance testing using the VITEK2 system (BioMérieux; Marcy-L’Étoile, France).

In the second study phase (1 July 2019–30 April 2020), the RDT was directly applied on the pellet from blood cultures within 2 h after they became positive for *Escherichia coli*, *K. pneumoniae* or *Pseudomonas aeruginosa*, as identified by the MALDI Sepsityper (Bruker Daltonics; Bremen, Germany). To this end, 1 ml of blood culture fluid was transferred to a microcentrifuge tube, 200 μl lysis buffer (MBT Sepsityper®, Bruker Daltonics; Bremen, Germany) was added, and the sample was thoroughly mixed. After centrifugation for 2 min, the supernatant was discarded, and 1 ml of wash buffer was added. The solution was mixed again and centrifuged for 1 min. Then, the pellet was mixed with 12 drops of the RDT’s LY-A buffer, and 3 drops were added to the sample wells of both RDT cassettes, as recommended in the manufacturer’s instructions.

Samples were included in this study when the patients had previously documented intestinal carriage of carbapenemase-producing bacteria or when there was an elevated likelihood for these pathogens (e.g. septic patient non-responsive to carbapenem treatment). Of note, the RDT results became available before comprehensive resistance testing results were reported.

During both phases of the study, RDT results were immediately communicated to the treating clinician and were jointly discussed, and alterations of the antibiotic treatment were documented.

Additional clinical data were analysed using patient chart review and documented notes from infectious disease consultations. No ethics approval was required for this study.

## Results

During the first study phase (12 months), the RDT was performed on a total of 29 clinical samples from 27 patients that were carbapenem-resistant on antimicrobial susceptibility testing. The test was most frequently used on isolates from urine samples (*n* = 11) and blood cultures (*n* = 8), followed by wound swabs (*n* = 5), catheter tips (*n* = 1), bronchoalveolar lavage (*n* = 1), cerebrospinal fluid (*n* = 1), abdominal drainage fluid (*n* = 1) and bile (*n* = 1). Bacterial species subjected to the RDT included *K. pneumoniae* (*n* = 19), *Klebsiella aerogenes* (*n* = 4), *Enterobacter cloacae complex* (*n* = 3), *Escherichia coli* (*n* = 1), *Serratia ureilytica* (*n* = 1) and *Serratia marcescens* (*n* = 1).

The RDT yielded a positive result in 6/27 tested patients (22%) and identified four KPC- and two OXA-48-producing *K. pneumoniae* strains, whereas no carbapenemases were detected in other bacterial species. All RDT results were confirmed by multiplex PCR, leading to a perfect diagnostic agreement between both tests. Details on the patient characteristics, clinical cases and the impact of RDT results on clinical management are provided in Table 1. In brief, carbapenemase detection led to an antibiotic treatment modification in all six patients, with a switch from meropenem-based regimens to ceftazidime/avibactam-containing regimens being most frequently performed. Microbiological cure and survival of the acute infection were achieved in five of these patients. One patient died, whose *K. pneumoniae* isolate was resistant to carbapenems, ceftazidime/avibactam, tigecycline, colistin and aminoglycosides.

**Table 1:** Microbiological and patient characteristics of invasive infections caused by carbapenemase-producing *Klebsiella pneumoniae* in a University hospital in Homburg, Germany (April 2018–April 2019)

Patient no.	Sex	Age (years)	Cause of hospitalization	Treating ward	Biological specimen	Carbapenemase detected	Impact of RDT on same-day antibiotic treatment decision?	Susceptible antibiotics on final testing report	Patient survived
1	Male	75	Mantle cell lymphoma	Haematology ICU	Blood culture	KPC-3	Yes (meropenem → ceftazidime/avibactam)	Ceftazidime/avibactam, colistin, gentamicin	Yes
2	Female	77	Ventriculitis	Neurosurgery ICU	Cerebrospinal fluid	KPC-3	Yes (meropenem + vancomycin → ceftazidime/avibactam + tigecycline)	Ceftazidime/avibactam, colistin, fosfomycin, gentamicin, tigecycline	Yes
3	Female	60	Peritonitis	General surgery	Abdominal drainage fluid	KPC-3	Yes (meropenem → ceftazidime/avibactam)	Ceftazidime/avibactam, colistin, gentamicin, tigecycline	Yes
4	Male	77	Acute kidney failure	Cardiology ICU	Blood culture, catheter tip, urine	OXA-48	Yes (no antibiotic treatment ➔ ceftazidime/avibactam)	Ceftazidime/avibactam, tigecycline, cotrimoxazole	Yes
5	Male	65	Decompensated heart failure	Cardiology ICU	Blood culture	OXA-48	Yes (meropenem + vancomycin ➔ ceftazidime/avibactam, later switch to tigecycline + cotrimoxazole)	Cotrimoxazole	No
6	Male	61	Acute myeloblastic leukaemia	Haematology ICU	Blood culture	KPC (not further specified)	Yes (no antibiotic treatment ➔ ceftazidime/avibactam)	Amikacin, ceftazidime/avibactam, colistin, fosfomycin	Yes

*ICU* intensive care unit

A rapid diagnostic test (RDT) was used to characterize carbapenemases, and the impact of the RDT result on a modification of the antibiotic treatment on the same day was reported

**Fig. 1:**
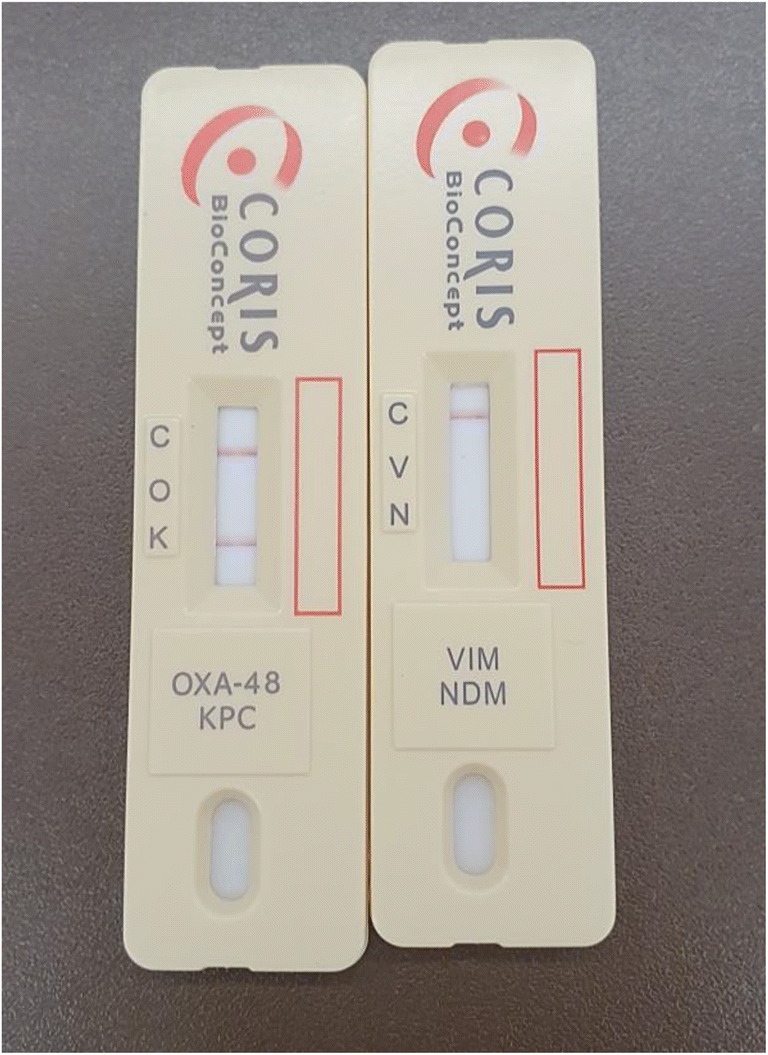
Test band intensity of the RESIST-4 O.K.N.V rapid diagnostic test for carbapenemase detection after direct application on the pellet of a positive blood culture. The shown test was performed on a blood culture with a KPC-positive *Klebsiella pneumoniae* strain. *C* control line, *O* OXA-48 carbapenemase, *K* KPC carbapenemase, *V* VIM carbapenemase, *N* NDM carbapenemase

In the second study phase (10 months), the RDT was applied on eleven positive blood culture samples stemming from eleven patients with an increased likelihood of carbapenemase-producing bacteria. The RDT was positive in 6/11 (55%) cases, all of which were identified as KPC-producing *K. pneumoniae* strains. Of note, RDT test band intensities were strong, and we did not observe faint or borderline test bands (Fig. 1). There was a 100% diagnostic agreement between the RDT carried out on blood culture pellets and multiplex PCR, which was performed the next day on culture-grown colonies. While three KPC-positive patients had previously documented intestinal carriage, the RDT was the first test being positive for carbapenemases in the other three patients. Treatment was modified in all patients from carbapenem-based to ceftazidime/avibactam-containing regimens, and all patients survived the bacteremia episode (Table 2).

**Table 2:** Microbiological and patient characteristics of bloodstream infections caused by *Klebsiella pneumoniae* carbapenemase (KPC)-producing *K. pneumoniae* in a University hospital in Homburg, Germany (July 2019–April 2020)

Patient no.	Sex	Age (years)	Cause of hospitalization	Treating ward	Intestinal carriage of KPC?	Impact of RDT on same-day antibiotic treatment decision?	Susceptible antibiotics on final testing report	Patient survived?
1	Male	67	Multiple myeloma	Haematology ICU	No	Yes (meropenem → ceftazidime/avibactam + colistin)	Amikacin, ceftazidime/avibactam, colistin, fosfomycin	Yes
2	Female	51	Acute myeloblastic leukaemia	Pneumology ICU	No	Yes (meropenem + vancomycin → ceftazidime/avibactam + colistin)	Amikacin, ceftazidime/avibactam, colistin	Yes
3	Male	77	Mantle cell lymphoma	Haematology ICU	No	Yes (meropenem + vancomycin → ceftazidime/avibactam + colistin)	Amikacin, ceftazidime/avibactam, colistin, fosfomycin	Yes
4	Male	66	CNS lymphoma	Haematology ICU	Yes	Yes (piperacillin/tazobactam + amikacin → ceftazidime/avibactam + amikacin)	Amikacin, ceftazidime/avibactam, colistin	Yes
5	Female	62	Acute myeloblastic leukaemia	Haematology ICU	Yes	Yes (piperacillin/tazobactam → ceftazidime/avibactam + colistin)	Amikacin, ceftazidime/avibactam, colistin	Yes
6	Female	64	Acute myeloblastic leukaemia	Haematology ICU	Yes	Yes (meropenem → ceftazidime/avibactam + colistin)	Amikacin, ceftazidime/avibactam, colistin	Yes

*ICU* intensive care unit

A rapid diagnostic test (RDT) was used directly on the pellet of blood cultures to characterize carbapenemases, and the impact of the RDT result on a same-day antibiotic treatment modification was documented. Of note, KPC-3 was identified in patients 1–4, while no further carbapenemase subtyping was performed in patients 5 and 6

## Discussion

The main findings of the present study are as follows: (i) in a clinical setting with a relatively low incidence of carbapenemases, the RESIST-4 O.K.N.V. test has excellent diagnostic accuracy, with reliable results obtained from culture-grown colonies and blood culture pellets, and (ii) immediate result communication to the treating clinician most frequently led to an early treatment modification, which likely resulted in an improved outcome. To our knowledge, our study is the first to report data on the test’s performance and clinical impact if applied to blood cultures from routine clinical practice with an elevated likelihood of carbapenemases (i.e. patients with a clinical deterioration during carbapenem treatment or known faecal carriage of carbapenemases). Our strategy frequently led to treatment modifications before final antimicrobial susceptibility tests became available and was based on the epidemiological likelihood of, for example, KPC-positive isolates being susceptible to ceftazidime/avibactam. Hence, this RDT may provide accurate, clinically relevant results faster and at a lower cost than PCR tests and might thus also be promising in resource-constrained settings with limited diagnostic infrastructure [6].

Having originally been designed to detect only one specific type of carbapenemases, the RDT used in this study was adapted several times to accommodate the concurrent detection of KPC and OXA-48 carbapenemases [7], with later addition of NDM [8] and VIM. In the meantime, an updated version was developed and made commercially available, which also includes OXA-163 [9]. Several laboratory assessments have shown excellent sensitivity and specificity, as elucidated by, for example, a detailed validation on clinical samples in the Belgian National Reference Centre for identification of the mechanisms related to carbapenem resistance [5]. In a recent study, the sensitivity and specificity of the RESIST-4 O.K.N.V. assay on culture-grown colonies were reported at 97.8% and 100%, respectively [10]. One study, conducted at the German National Reference Laboratory for multidrug-resistant gram-negative bacteria, used the RDT to analyse 100 clinical carbapenemase isolates and showed a 100% agreement between the RDT results and molecular reference testing [11]. Of note, more recent investigations observed a slightly reduced test sensitivity for detection of NDM carbapenemases [12], which were absent in our study, and decreased accuracy if the RDT was performed on bacterial colonies from Mueller-Hinton agar as compared with sheep blood agar [13].

A rapid diagnosis of carbapenemase-producing bacteria is essential to guide clinical decision-making. The RDT used in this study was designed to be performed on culture-grown colonies on agar plates. However, previous studies employed different versions of this RDT as a screening method for faecal carriage of multiresistant bacteria directly on rectal swabs [4], with comparable diagnostic accuracy if compared with culture [14]. With regard to positive blood culture bottles, a proof-of-principle study conducted on the previous version of this RDT (RESIST-3) showed a sensitivity and specificity of 100%, respectively, for carbapenemase detection if blood cultures were spiked in the laboratory with carbapenemase-producing bacteria and the test applied on a centrifuged lysate of positive blood cultures [15]. Of note, an additional evaluation from Italy on spiked blood cultures elucidated that a newer version of the RDT (RESIST-5) failed to detect KPC variants with a D179Y point mutation and observed a lower sensitivity for detection of VIM and NDM on blood culture pellets [9], which is in line with findings from a previous study [16]. The D179Y point mutation may confer resistance to ceftazidime-avibactam [17].

Our study has some limitations. First, the sample size in our exploratory single-centre study was small, and there is a need for larger, prospective studies to confirm the clinical utility of our approach. Second, we did not consistently use a specific grading system to document the RDT’s test band intensity, which is reported to vary across clinical samples [18] and ‘trace’ results might pose a diagnostic challenge. Third, we only evaluated one RDT, the RESIST-4 O.K.N.V., on samples positive for OXA-48 and KPC, whereas other RDTs have shown comparable diagnostic accuracy [19]. Fourth, the emerging resistance to ceftazidime/avibactam, which was frequently used in our study for the treatment of carbapenemase-producing bacteria, might limit the utility of our approach [20]. Indeed, we observed one *K. pneumoniae* strain resistant to ceftazidime/avibactam, but did not further specify the underlying resistance mechanism. This should be addressed in future investigations. However, this development might be counterbalanced by the introduction of new antibiotics such as cefiderocol or meropenem/vaborbactam, which might be suitable alternatives for infections caused by bacteria with OXA-48 or KPC carbapenemases.

In conclusion, the RDT used in this study showed excellent diagnostic accuracy to detect KPC and OXA-48 carbapenemases in clinical samples. The RDT provided reliable results in less than 30 min, both on culture-grown colonies as well as on pellets from positive blood cultures. Most importantly, carbapenemase detection always led to a treatment modification—frequently before additional antibiotic testing results became available—and might have improved patient outcome. Further studies are warranted to investigate the effects of such rapid diagnostics and close collaboration between clinical and diagnostic infectious disease disciplines to improve patient management.
